# Hospital Needs

**Published:** 1912-11-23

**Authors:** 


### Hospital Needs.

### THE FINE ART OF ORTHOPÆDIC MECHANICS.

THE recent increased attention given in this country to orthopædics, with the development and extension of the special departments devoted to its theory and practice by the hospitals, gives an added interest to the new catalogue of artificial limbs and eyes issued by Mr. W. R. Grossmith, 110 Strand, London, W.C. This house already has made its specialty accuracy of fitting, lightness and strength, and there are few hospital managers who cannot learn something from its parallel accounts of special appliances and the experience of patients who have used them. These two sections illustrate each other in a very striking fashion. Illustrations are given of limbs for amputations at the hip joint, above, at, or below the knee, at the ankle joint, for Chopart's amputation, and so on, in addition to those of artificial hands and arms. Space unfortunately precludes an examination of detail, but the main facts can be repeated, that for accuracy of finish, lightness, and durability combined these models cannot probably be surpassed anywhere. Mr. Grossmith, while entering deeply into this question, has not fallen into the other extreme of evolving an absurd complexity of structure, which is an error only the finest skill can avoid.

